# GSDMD gene knockout alleviates hyperoxia-induced hippocampal brain injury in neonatal mice

**DOI:** 10.1186/s12974-023-02878-8

**Published:** 2023-09-07

**Authors:** Naga Venkata Divya Challa, Shaoyi Chen, Huijun Yuan, Matthew R. Duncan, William Javier Moreno, Helen Bramlett, W. Dalton Dietrich, Merline Benny, Augusto F. Schmidt, Karen Young, Shu Wu

**Affiliations:** 1 Department of Pediatrics/Division of Neonatology, Batchelor Children’s Research Institute, Holtz Children’s Hospital, University of Miami Miller School of Medicine, Miami, FL USA; 2 Miami Project to Cure Paralysis and Department of Neurological Surgery, University of Miami Miller School of Medicine, Miami, FL USA

**Keywords:** GSDMD knockout, Neonatal, Brain injury, Pyroptosis, Microglial, Gene transcription

## Abstract

**Background:**

Neonatal hyperoxia exposure is associated with brain injury and poor neurodevelopment outcomes in preterm infants. Our previous studies in neonatal rodent models have shown that hyperoxia stimulates the brain’s inflammasome pathway, leading to the activation of gasdermin D (GSDMD), a key executor of pyroptotic inflammatory cell death. Moreover, we found pharmacological inhibition of caspase-1, which blocks GSDMD activation, attenuates hyperoxia-induced brain injury in neonatal mice. We hypothesized that GSDMD plays a pathogenic role in hyperoxia-induced neonatal brain injury and that GSDMD gene knockout (KO) will alleviate hyperoxia-induced brain injury.

**Methods:**

Newborn GSDMD knockout mice and their wildtype (WT) littermates were randomized within 24 h after birth to be exposed to room air or hyperoxia (85% O_2_) from postnatal days 1 to 14. Hippocampal brain inflammatory injury was assessed in brain sections by immunohistology for allograft inflammatory factor 1 (AIF1) and CD68, markers of microglial activation. Cell proliferation was evaluated by Ki-67 staining, and cell death was determined by TUNEL assay. RNA sequencing of the hippocampus was performed to identify the transcriptional effects of hyperoxia and GSDMD-KO, and qRT-PCR was performed to confirm some of the significantly regulated genes.

**Results:**

Hyperoxia-exposed WT mice had increased microglia consistent with activation, which was associated with decreased cell proliferation and increased cell death in the hippocampal area. Conversely, hyperoxia-exposed GSDMD-KO mice exhibited considerable resistance to hyperoxia as O_2_ exposure did not increase AIF1 + , CD68 + , or TUNEL + cell numbers or decrease cell proliferation. Hyperoxia exposure differentially regulated 258 genes in WT and only 16 in GSDMD-KO mice compared to room air-exposed WT and GSDMD-KO, respectively. Gene set enrichment analysis showed that in the WT brain, hyperoxia differentially regulated genes associated with neuronal and vascular development and differentiation, axonogenesis, glial cell differentiation, hypoxia-induced factor 1 pathway, and neuronal growth factor pathways. These changes were prevented by GSDMD-KO.

**Conclusions:**

GSDMD-KO alleviates hyperoxia-induced inflammatory injury, cell survival and death, and alterations of transcriptional gene expression of pathways involved in neuronal growth, development, and differentiation in the hippocampus of neonatal mice. This suggests that GSDMD plays a pathogenic role in preterm brain injury, and targeting GSDMD may be beneficial in preventing and treating brain injury and poor neurodevelopmental outcomes in preterm infants.

**Supplementary Information:**

The online version contains supplementary material available at 10.1186/s12974-023-02878-8.

## Introduction

Each year more than 15 million infants are born preterm worldwide [1]. Extremely premature infants born at less than 28 weeks of gestational age are at great risk of having a multi-organ injury that predominantly involves the lung and brain [2–5]. Born with immature lungs, these premature infants suffer respiratory failure soon after birth and often require oxygen (O_2_) therapy and mechanical ventilation to survive. However, life-sustaining high-concentration O_2_ therapy (hyperoxia) can cause lung inflammation that ultimately leads to bronchopulmonary dysplasia (BPD), characterized by disrupted alveolar and vascular development and reduced lung function [2, 3]. The immature brains in these premature infants are also affected by the hyperoxia that results in inflammation leading to short-term and long-term neurodevelopmental sequelae, such as intraventricular hemorrhage, encephalopathy of prematurity, cerebral palsy, intellectual disability, and cognitive deficits [4, 5]. Therefore, survivors of BPD are known to suffer not only from long-term lung disease but also suffer from long-term sequelae involving the brain leading to long-term neurodevelopmental impairment (NDI). Moreover, there is mounting clinical evidence that severe BPD is an independent risk factor for adverse neurodevelopmental outcomes, even without catastrophic brain injury [5–7]. Furthermore, many preclinical studies using rodent models also support the critical role of neonatal hyperoxia in inducing various injuries in developing brains [8–10].

Inflammasomes are large macromolecular signaling complexes that, when activated by pathogens or host-derived danger signals, lead to the activation of the inflammatory caspases, which in turn control the proteolytic activation of two highly proinflammatory IL-1 family cytokines, IL-1β and IL-18 [11–14]. Stimulation of these inflammasome cascades also activates gasdermin D (GSDMD), a 53-kilodalton (kDa) cytosolic protein, which has been recently found to be a key executor of pyroptosis, a form of programmed inflammatory cell death [11–14]. GSDMD is cleaved by inflammasome-associated inflammatory caspases 1/4/5 in humans and 1/11 in rodents, which releases a 30-kDa N-terminal domain (p30) that oligomerizes in the cell membrane to form pores, which cause localized cellular swelling, membrane rupture, and cell death, known as pyroptosis. In addition, the pores formed by GSDMD-p30 oligomerization also allow rapid release of active IL-1β and IL-18, resulting in secondary inflammation. Growing evidence shows activation of the inflammasome cascade in the lung and brain secondary to hyperoxia [15–17]. Previous studies have demonstrated early activation of the NLRP3 inflammasome with an increased IL1β:IL1ra ratio is a key mechanism in the development of BPD [18, 19]. Many studies have demonstrated a critical role for GSDMD in regulating pyroptosis and inflammation in various adult diseases. Recent studies from our laboratory have highlighted the crucial role of GSDMD in hyperoxia-induced and mechanical ventilation-associated neonatal lung and brain injury in rodent models [15, 16, 20]. Most recently, we have demonstrated that GSDMD gene knockout (KO) [11] ameliorated hyperoxia-induced BPD and retinopathy of prematurity (ROP) in mouse models [21], indicating a critical role for GSDMD in hyperoxia-induced preterm multi-organ injury.

In this study, we hypothesized GSDMD-KO would alleviate hyperoxia-induced brain injury in neonatal mice. To test this hypothesis, we utilized global GSDMD-KO mice [11] and their wildtype (WT) littermates and exposed them to 85% O_2_ from postnatal days (P) 1 to P14. We found that GSDMD-KO reduced hyperoxia activation of microglia and cell death and improved cell survival in the hippocampal area. We also performed RNA sequencing (RNA-seq) analyses of the hippocampus and found that GSDMD-KO prevented hyperoxia induction of genes involved in neuron differentiation and development, synapse assembly, axonogenesis, and vascular development. These findings not only fill a gap in understanding the critical role of GSDMD in the pathogenesis of hyperoxia-induced brain injury but also identify potential novel targets for preventing and treating brain injury in premature infants.

## Methods

### Materials

Please see Additional file 1 for material lists.

### Animals and study approval

The Animal Care and Use Committee of the University of Miami Miller School of Medicine approved the experimental protocol. All animals were cared for according to the National Institutes of Health guidelines for the use and care of animals. The study is reported in accordance with ARRIVE. GSDMD-KO mice (C57BL/6N) [9] were obtained from Jackson Laboratory (Bar Harbor, ME). Heterozygote female and male mice were mated to produce newborn mice. Tail biopsy was done on newborn mice at P7 for DNA extraction and PCR with primers to identify WT mice and homozygous KO mice which carry CRISPR/Cas9-derived knockout alleles that incorporate a 38 bp deletion in exon 5 of the GSDMD gene (11). Experiments were done with homozygous KO mice and their WT littermates.

### Hyperoxia-induced BPD model

Newborn GSDMD-KO mice and their WT littermates were exposed to RA (21% O_2_) or hyperoxia (85% O_2_) from P1 to P14, as previously described [15].

### Hippocampal tissue collection and brain tissue section

On P15, the pups were anesthetized by 0.1% isoflurane, and their hippocampal tissues were collected and frozen in − 80° C for RNA isolation. Their brain tissues were fixed in 10% formalin, paraffin-embedded, and cut serially using a calibrated rotary microtome into 10 μm coronal sections after removal of the olfactory and frontal poles.

### Assessment of GSDMD expression in specific cells of hippocampal tissues

Brain tissue sections were immunostained with an anti-GSDMD antibody to determine GSDMD protein expression. Double immunofluorescent staining was performed with anti-GSDMD and antibodies for specific brain cells, including AIF1 (microglial cells) [22], glial fibrillary acidic protein (GFAP, astrocytes) [23], neuronal nuclei (NeuN, neurons) [24], sex-determining region Y-related HMG box 2 (SOX2, neural stem cells and progenitor cells) [25].

### Assessment of hippocampal inflammation

Microglial infiltration was determined by immunostaining with an anti-AIF1 antibody and an anti-CD68 antibody [22]. The number of AIF1-stained and CD68-stained cells in the hippocampal sections was counted from 5 random high-power views (HPV) taken from the 20X objective on each slide [16]. Total RNA was extracted from frozen hippocampal tissues using the RNeasy Universal Mini Kit (Qiagen, Valencia, CA) according to the manufacturer’s instructions. RNA quality and integrity were verified using the Agilent 2100 Bioanalyzer (Agilent Technologies, Santa Clara, CA). All samples had RNA integrity numbers > 7. Gene expression of inflammatory mediators, *Il1β*, *Il1r1*, *Il18*, *II6*, and *Il33* was determined by real-time qRT-PCR, which was performed on an ABI Fast 7500 System (Applied Biosystems, Foster City, CA) as previously described [16]. The expression levels of target genes were normalized to 18S rRNA.

### Assessment of hippocampal cell proliferation and death

Cell proliferation was assessed by immunofluorescent staining for Ki67, a nuclear proliferation marker, and the proliferating index was calculated as the average percentage of Ki67-positive nuclei in total nuclei in 5 random HPV on hippocampal sections from each animal. Cell death was studied using a TUNEL assay, and the cell death index was calculated as the average percentage of TUNEL-positive nuclei in total nuclei in 5 random HPV on hippocampal sections from each animal [15, 19]. Immunofluorescent staining for neural cell-specific markers, AIF1, GFAP, NeuN, and SOX2, was performed with a TUNEL assay to co-localize TUNEL-positive nuclei in specific neural cells.

### RNA-seq

RNA-seq was performed by BGI Genomics (Hong Kong) with a read depth of 30 million reads per sample for 150 bp paired-end reads. The raw sequence read in FASTQ format was aligned to the mouse (*Mus musculus*) genome build mm_GRCm39_104 using Kallisto [26], followed by gene summarization with tximport [27]. After checking data quality, differential expression analyses comparing treatment groups to control and to each other were performed using DESeq2 with false discovery adjustment [28]. Genes were considered differentially expressed based on their fold-change relative to control (= or > 1.25), *P* value (< 0.05), and *q* value (< 0.1) [29]. Lists of differentially expressed genes were used for functional enrichment analysis of Gene Ontology and pathway terms using the ToppCluster [30]. Only unique terms associated with either induced or suppressed genes and at least 2 genes were reported [31].

### Data management and statistical analysis

Data were expressed as mean ± SD, and comparisons between groups were performed using one-way ANOVA followed by Turkey’s post-hoc analysis. A *P* value of 0.05 was considered significant.

## Results

### GSDMD expression in the hippocampus

**Fig. 1:**
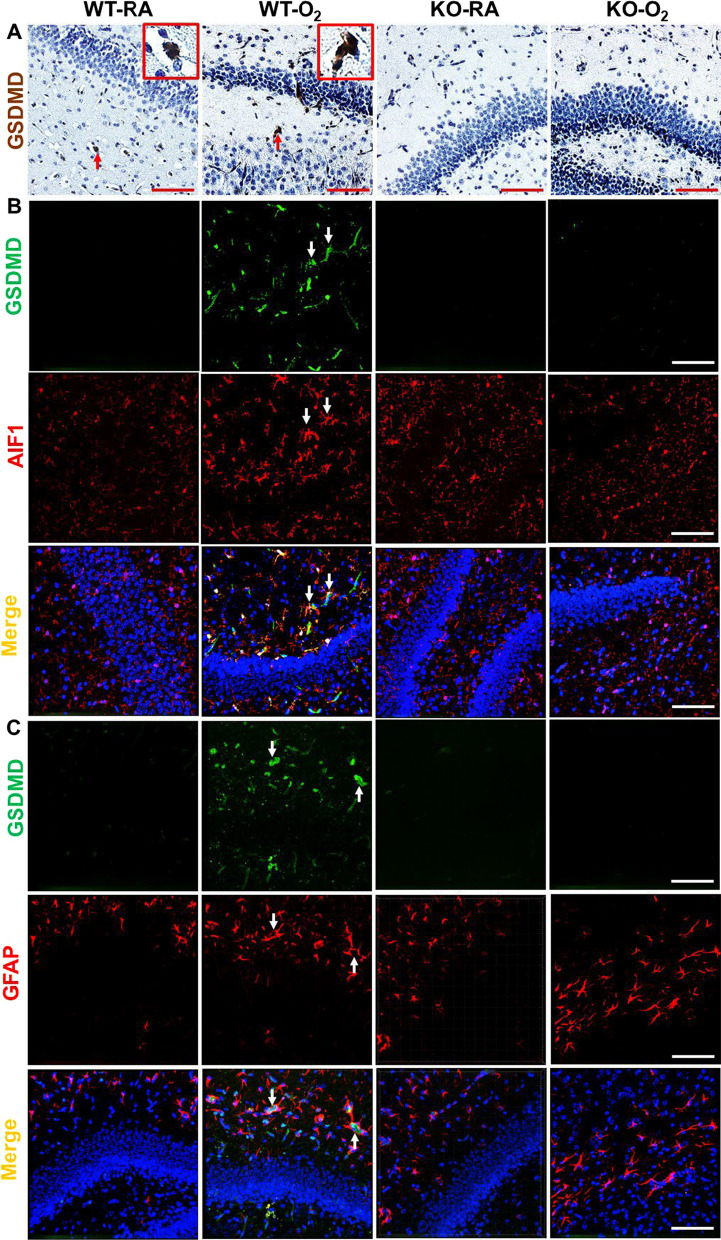
GSDMD expression in the brains of the four study groups. **A** GSDMD immunostaining showed GSDMD expression was increased in the hippocampal area of hyperoxia-exposed wildtype (WT-O_2_) brain (red arrows) compared to the hippocampal areas of the room air-exposed WT (WT-RA), room air-exposed GSDMD-KO (KO-RA), and hyperoxia-exposed GSDMD-KO (KO-O_2_) hippocampi. Representative focal enlarged areas of GSDMD + cells are in the red boxes. 20 × objective magnification. Scale bar: 50 µm. **B** Double immunofluorescent staining for GSDMD (green signal), AIF1 (red signal), and DAPI (blue signal, not shown). GSDMD was co-localized with AIF1 in WT-O_2_ hippocampus (orange signals, white arrows). Scale bar: 50 µm. **C** Double immunofluorescent staining for GSDMD (green signal), GFAP (red signal), and DAPI (blue signal, not shown). GSDMD was co-localized with GFAP in the WT-O_2_ hippocampus (orange signals, white arrows). Scale bar: 50 µm

We first showed that GSDMD is expressed in the room air-exposed WT (WT-RA) hippocampal sections, and it was increased in the hyperoxia-exposed WT (WT-O_2_) hippocampal sections. However, GSDMD was undetectable in RA-exposed GSDMD-KO (KO-RA) brains and hyperoxia-exposed GSDMD-KO (KO-O_2_) brains (Fig. 1A). These results confirmed the absence of GSDMD in the KO brains, and that hyperoxia increases GSDMD protein expression in the WT hippocampus. GSDMD gene expression measured by qRT-PCR also showed GSDMD-KO mice brains lacked measurable GSDMD transcripts (data not shown). To determine the specific brain cells that express GSDMD, we performed double immunofluorescence of GSDMD with cell-specific markers, including AIF1, GFAP, NeuN, and SOX2. GSDMD was co-localized with AIF1, suggesting GSDMD is expressed by microglial cells (Fig. 1B). GSDMD was also co-localized with GFAP, indicating GSDMD is expressed by astrocytes (Fig. 1C). GSDMD was rarely co-localized with SOX2 but never co-localized with NeuN, suggesting GSDMD is weakly expressed by neural stem cells and progenitor cells, but not by neurons (data not shown).

### GSDMD-KO reduces hippocampal inflammation in hyperoxia-exposed neonatal mice

**Fig. 2:**
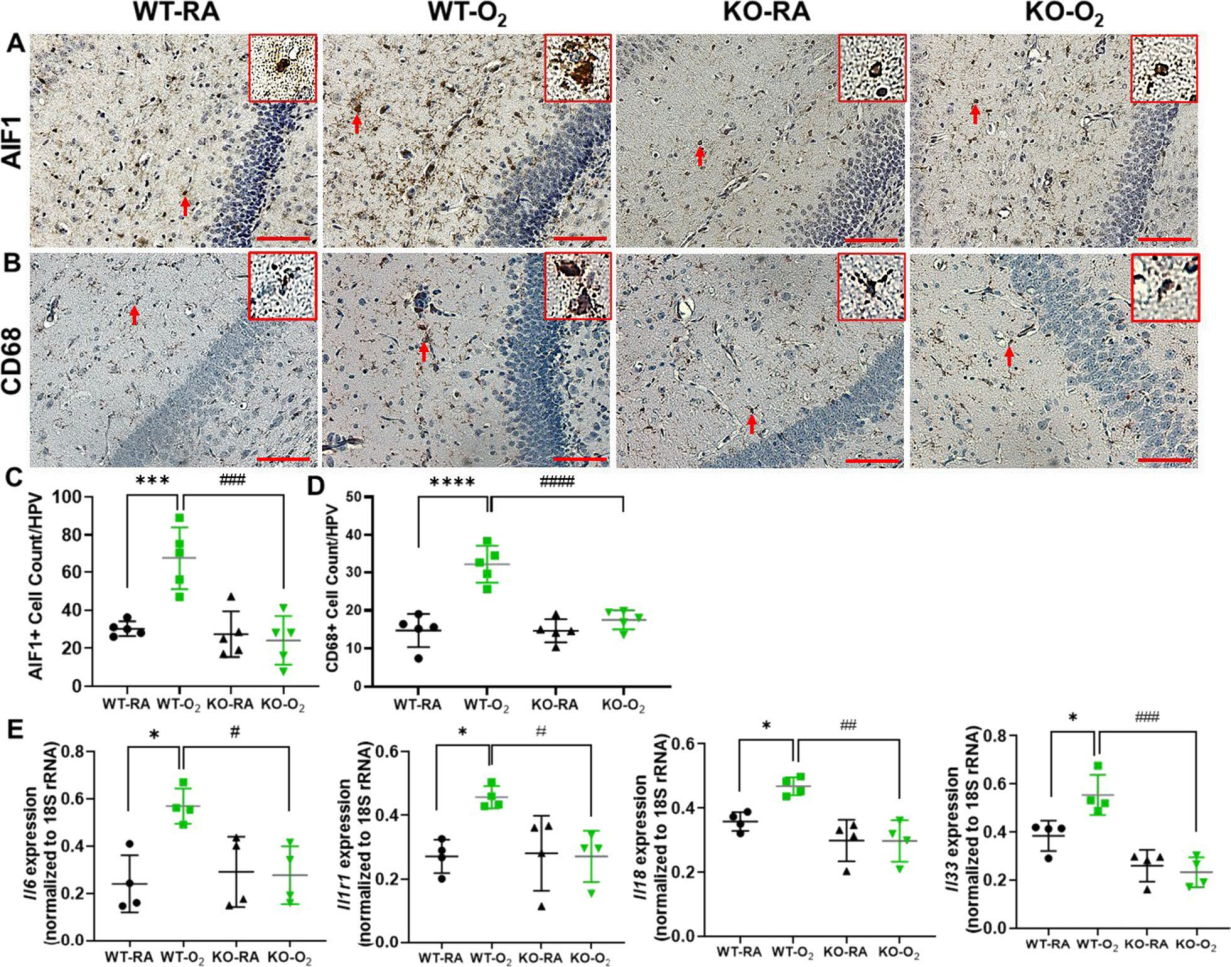
GSDMD-KO reduces hyperoxia-induced hippocampal inflammation. **A** Immunostaining for AIF1 (a microglial marker, brown signals, red arrows) (**A**) and immunostaining for CD68 (a microglial marker, brown signals, red arrows) (**B**) showed microglia cells in the WT-O_2_ hippocampus were disorganized and had enlarged bodies and dendrites compared to hippocampi from WT-RA, KO-RA, and KO-O_2_ mice. Representative focal enlarged areas of microglial cells are in the red boxes. There was a significant increase of AIF1 + microglial cells (**C**) and CD68 + microglial cells (**D**) in the WT-O_2_ group compared to the WT-RA group, but KO-O_2_ had reduced microglial cells compared to the WT-O_2_ group. n = 5/group. ***P < 0.001 and ****P < 0.0001, WT-RA vs. WT-O_2_. ^###^*P* < 0.001 and ^####^*P* < 0.0001, WT-O_2_ vs. KO-O_2_. 20 × objective magnification. Scale bars: 50 µm. **E** qRT-PCR demonstrated increased gene expression of inflammatory mediators, including *Il6*, *Il1r1*, *Il18*, and *Il33,* in WT-O_2_ brains compared to WT-RA brains. However, KO-O_2_ mice had reduced expression of these genes compared to WT-O_2_ brains. n = 4/groups. *P < 0.05 and ***P < 0.001, WT-RA vs. WT-O_2_. ^#^*P* < 0.05, ^##^*P* < 0.01 and ^###^*P* < 0.001, WT-O_2_ vs. KO-O_2_

We next examined hippocampal sections for microglial infiltration by immunostaining to assess whether GSDMD-KO affects hyperoxia-induced hippocampal inflammation. Histologically, there were many microglial cells that were positive for AIF1 and CD68 in the WT-O_2_ brains compared to the other three groups (Fig. 2A, B). These cells had activated microglia features, such as enlargement of the cell body, reduction in the territory, irregular cell shape, and self-association for each other (red boxes, Fig. 2A, B). Quantitative analysis showed that the AIF1 + microglial count was threefold higher in the WT-O_2_ group than the other three groups (*P* < 0.001, Fig. 2C). Similarly, the CD68 + microglial count was twofold higher than the other three groups (*P* < 0.0001, Fig. 2D). Thus, GSDMD-KO ameliorated hyperoxia-induced microglial cell activation in the hippocampus. We further assessed inflammation by qRT-PCR for gene expression of inflammatory mediators and demonstrated that hyperoxia increased expression of *Il6*, *Il1r1*, *Il18*, and *Il33*, but not *Il1β* in WT brains compared to room air-exposed WT brains. Moreover, hyperoxic exposure failed to increase the expression of any of these inflammation-associated genes in GSDMD-KO hippocampi (Fig. 2E). These data further support that GSDMD-KO reduces hyperoxia-induced brain inflammation.

### GSDMD deficiency improves cell survival and decreases cell death in hyperoxia-exposed brains

**Fig. 3:**
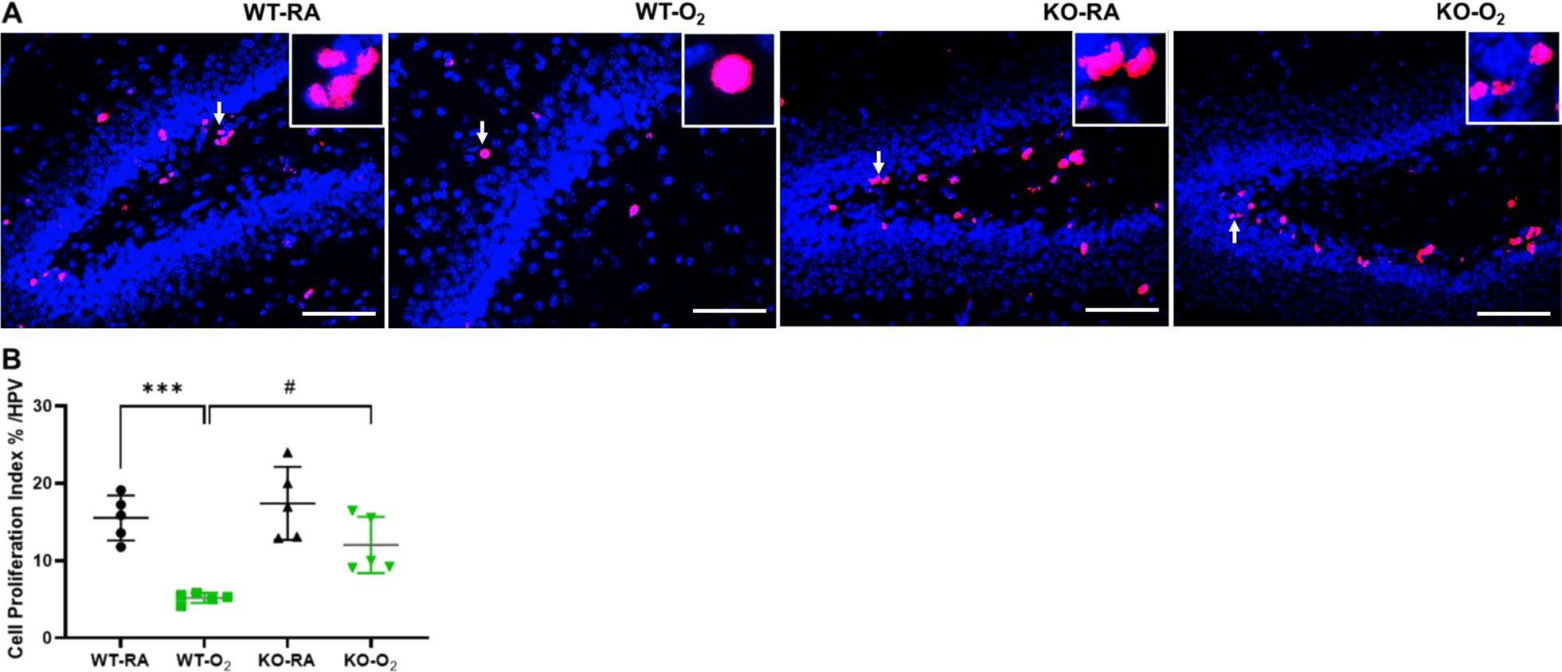
GSDMD-KO improves cell survival in hyperoxia-exposed brains. **A** Representative immunofluorescence staining for Ki67 (pink signals, red arrows) and DAPI nuclear staining (blue signals) in brain tissue sections from WT-RA, WT-O_2_, KO-RA, and KO-O_2_ mice. Representative focal enlarged areas of Ki67 + stained cells are in the white boxes. **B** Quantification of proliferation index (percentage of Ki67 + nuclei/total cell nuclei) showed a decreased Ki67 + cells in the WT-O_2_ group. In contrast, the GSDMD-KO group exposed to hyperoxia had increased Ki67 + cells compared to the WT-O_2_ group. n = 5/group. ***P < 0.001, WT-RA vs. WT-O_2_. ^#^*P* < 0.05, WT-O_2_ vs. KO-O_2_. 20 × objective magnification. Scale bars: 50 µm

**Fig. 4:**
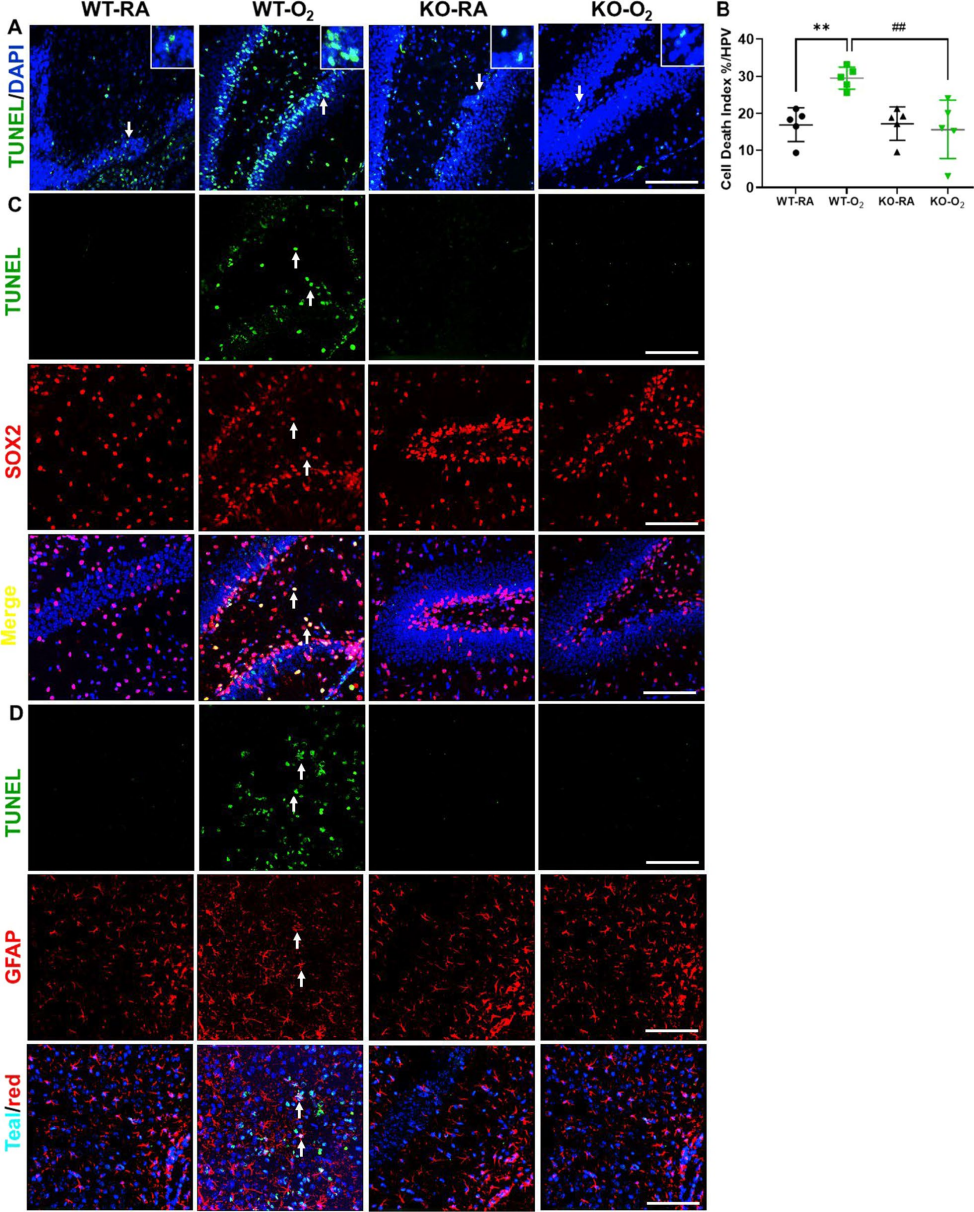
GSDMD-KO prevents cell death induced by hyperoxia. **A** TUNEL assay (green signals) and DAPI nuclear stain (blue signals) were used to identify dead cell nuclei (teal signals). Representative focal enlarged areas of TUNEL + stained cells were in the white boxes. **B** Quantification of cell death index (percentage of apoptotic nuclei divided by total nuclei) revealed that WT hippocampus had increased cell death when exposed to hyperoxia. In contrast, hyperoxia-exposed KO hippocampus had significantly less cell death. n = 5/group. **P < 0.01, WT-RA vs. WT-O_2._^##^*P* < 0.001, WT-O_2_ vs. KO-O_2._ 20 × objective magnification. Scale bars: 50 μm. **C** TUNEL assay (green signal) was performed with immunofluorescence for SOX2 (red signal), and DAPI (blue signal, not shown). TUNEL-positive nuclei were co-localized in SOX2-positive cells in WT-O_2_ hippocampus (white/yellow nuclei, white arrows). Scale bar: 50 µm. **D** TUNEL (green signal) was performed with immunofluorescence for GFAP (red signal), and DAPI (blue signal, not shown), TUNEL-positive nuclei were co-localized in GFAP-positive cells in WT-O_2_ hippocampus (teal nuclei/red cells, white arrows). Scale bar: 50 µm

GSDMD is a key executor of inflammasome-induced pyroptosis, and hyperoxia is known to reduce cell survival and cause cell death in hyperoxia-induced brain injury models. We found that the WT-O_2_ group showed a 67% decrease in cell proliferation compared to WT-RA (*P* < 0.01). However, the KO-O_2_ group had approximately 2.4 folds increased cell proliferation compared to WT-O_2_ group (*P* < 0.05, Fig. 3A, andB). When we assessed cell death, our data showed that the WT-O_2_ group had a nearly 50% increase in cell death compared to the other three groups (*P* < 0.01, Fig. 4A, andB). We further assessed which brain cells undergo cell death by co-localizing TUNEL-positive nuclei with cell-specific markers. As demonstrated in Fig. 4C, TUNEL-positive nuclei were mainly co-localized with SOX2-positive cells, indicating these are neural stem cells and progenitor cells. In addition, TUNEL-positive nuclei were also co-localized with GFAP-positive cells (Fig. 4D), suggesting these are astrocytes. TUNEL-positive nuclei were rarely co-localized with AIF1-positive cells (data not shown). Thus, hyperoxia induced cell death in neural stem cells, progenitor cells, astrocytes, and rarely in microglial cells.

### GSDMD deficiency ameliorates hyperoxia modulation of developmental processes and pathways in the hippocampus

**Fig. 5:**
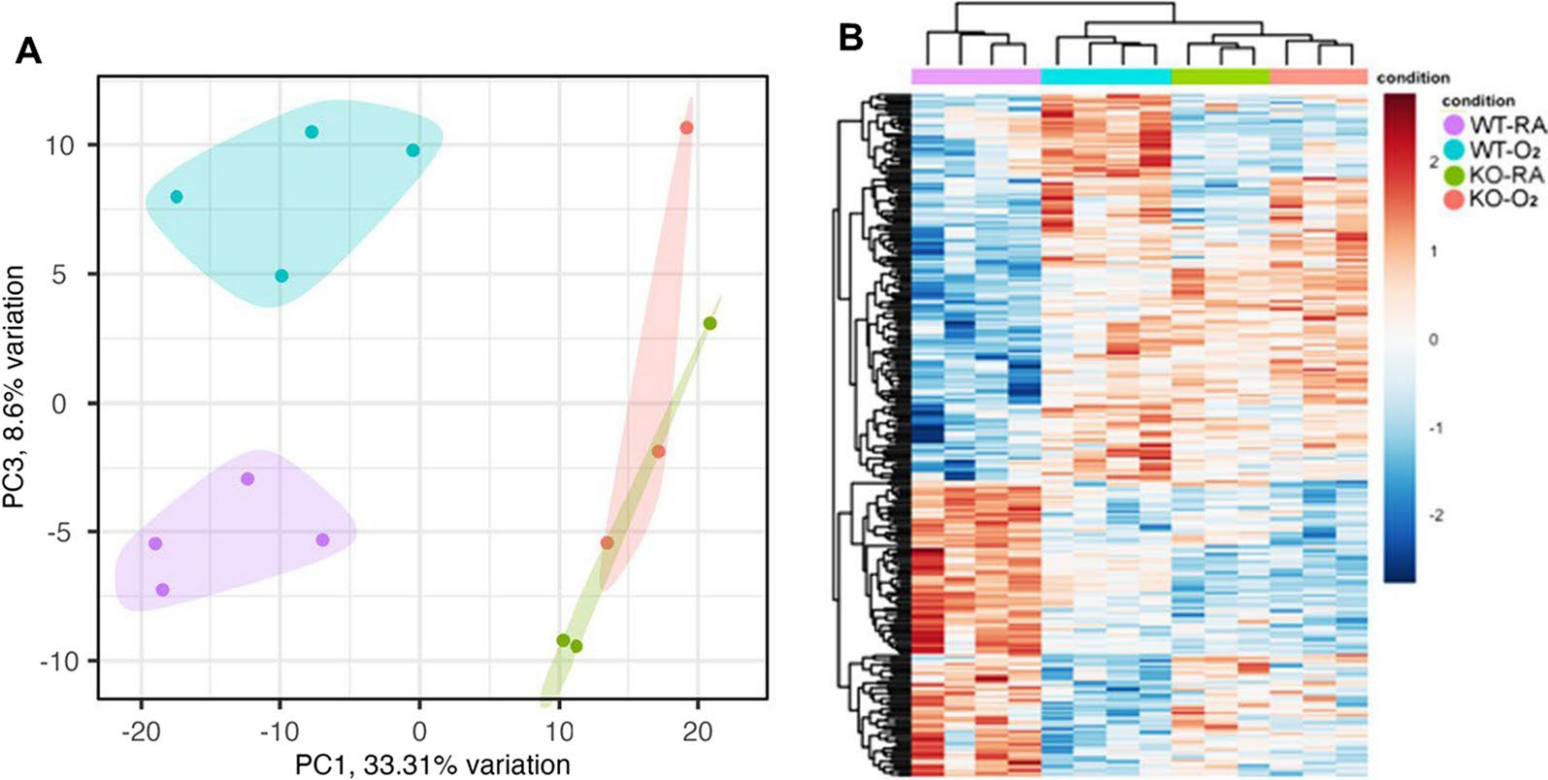
GSDMD-KO prevents transcriptional changes induced by hyperoxia in the hippocampus. **A** Principal component analysis (PCA) plot showing separation of WT and GSDMD-KO mice by PC1 and WT-RA and WT-O_2_ animals by PC3, but no separation between KO-O_2_ and KO-RA. **B** Heatmap of differentially expressed genes in WT-O_2_ vs. WT-RA and KO-O_2_ vs. KO RA with hierarchical clustering of treatment groups. n = 4 animals/group in WT groups. n = 3 animals/group in KO groups

We performed RNA-seq analysis to understand how GSDMD-KO affects the transcriptional response to hyperoxia. Principal component analysis showed clear separation of WT and KO animals by principal component 1 (PC1). In WT animals, PC3 separated WT-RA and WT-O_2_, but not KO-RA and KO-O_2_, which were not separable by any of the top 10 PCs (Fig. 5A). Heatmap of differentially expressed in WT-O_2_ vs. WT-RA and KO-O_2_ vs. KO-RA and hierarchical clustering of genes and samples showed clear clustering of samples by genetic backgrounds and conditions (Fig. 5B).

**Fig. 6:**
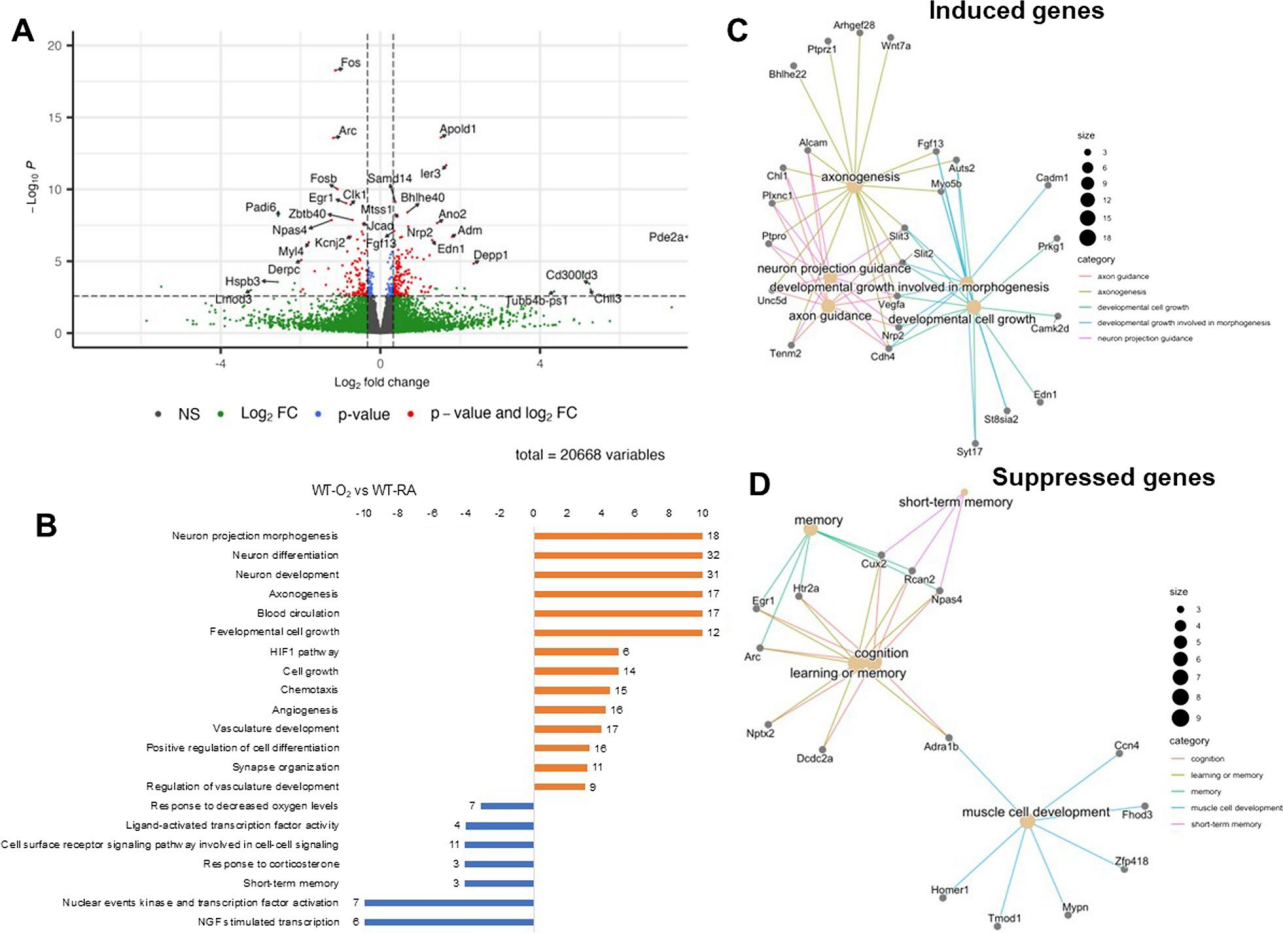
Hyperoxia modulates developmental pathways in the hippocampus of WT animals. **A** Volcano plot of genes differentially expressed in the hippocampus of room air-exposed WT mice compared to hyperoxia-exposed WT mice (fold change > 1.25 and FDR < 0.1). **B** Gene set enrichment analysis for Gene Ontology term and KEGG pathways of genes differentially expressed by hyperoxia-exposed WT mice. Hyperoxia-induced genes were associated with neuroprojection morphogenesis, neuron differentiation, neuron development, axonogenesis, blood circulation, HIF-1 pathway, cell growth, chemotaxis, angiogenesis, and vascular development. differentiation, development, and axonogenesis. Hyperoxia-suppressed genes were associated with NGF stimulated transcription, memory, nuclear events kinase and transcription factor activation, short-term memory, response to corticosterone, cell surface receptor signaling pathway involved in cell–cell signaling, ligand-activated transcription factor activity, and response to hypoxia. **C** Network plot of select top biological processes and their associated genes induced by hyperoxia. **D** Network plot of select top biological processes and their associated genes suppressed by hyperoxia

**Fig. 7:**
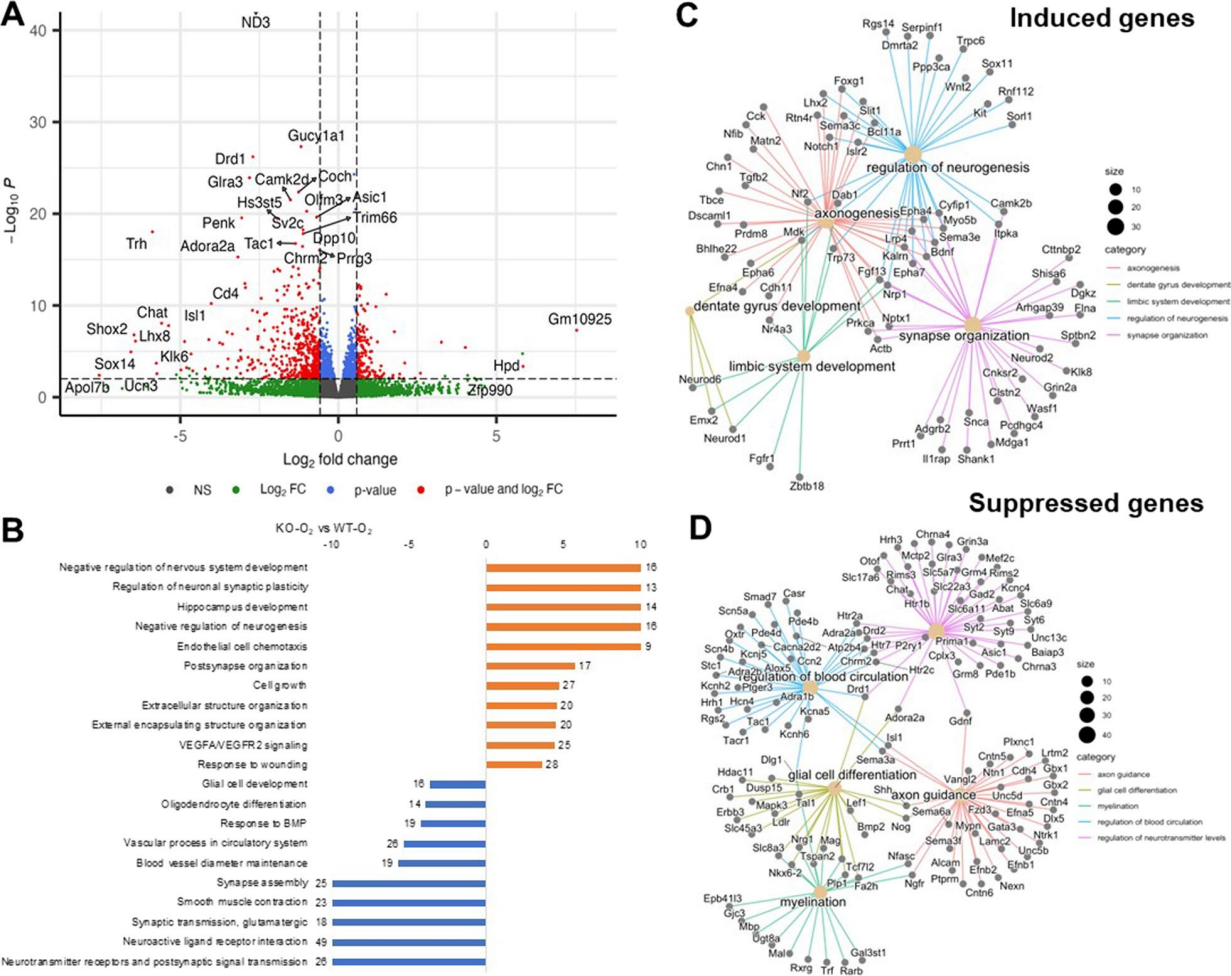
GSDMD KO modulates the transcriptional response to hyperoxia in the hippocampus. **A** Volcano plot of genes differentially expressed in brains of KO-O_2_ mice compared to WT-O_2_ mice (fold change > 1.25 and FDR < 0.1). **B** Gene set enrichment analysis of genes differentially expressed in hyperoxia-exposed KO mice compared to hyperoxia-exposed WT mice. In the KO-O_2_ group, there was an induction of genes associated with negative regulation of nervous system development, regulation of neuronal synaptic plasticity, hippocampus development, negative regulation of neurogenesis, endothelial cell chemotaxis, postsynapse organization, extracellular structure organization, and VEGFA/VEGFR2 signaling compared to WT-O_2_ group. Hyperoxia-suppressed gene pathways were associated with neurotransmitter receptors and postsynaptic signal transmission, neuroactive ligand receptor interaction, synaptic transmission, synapse asseembly, blood vessel diameter maintenance, vascular process in circulatory system, reponse to BMP, and oligodendrocyte differentiation. **C** Network plot of select top biological processes and their associated genes induced in KO-O_2_ compared to WT-O_2_. **D** Network plot of select top biological processes and their associated genes suppressed in KO-O_2_ compared to WT-O_2_ brains. n = 4 animals/group in WT groups. n = 3 animals/group in KO groups

We then performed differential expression analysis comparing WT-O_2_ vs. WT-RA hippocampus and KO-O_2_ vs. KO-RA hippocampus. In WT animals, hyperoxia differentially regulated 258 genes with 146 genes upregulated and 112 genes downregulated (Fig. 6A), whereas in GSDMD-KO animals, hyperoxia differentially regulated only 16 genes (Fig. 7A, B). Histogram of *P* values in KO-O_2_ vs. KO-RA showed uniform distribution, suggesting the few differentially expressed genes identified are likely false discoveries. We performed an overrepresentation analysis on Topcluster to identify biological processes and pathways for the genes induced and suppressed by hyperoxia in WT animals. The bar graph in Fig. 6B shows the top Gene Ontology Biological Processes and KEGG and Reactome pathways associated with genes induced and suppressed by hyperoxia in WT animals. Genes induced by hyperoxia were associated with neuroprojection morphogenesis, neuron differentiation, neuron development, axonogenesis, blood circulation, hypoxia-induced factor 1 (HIF-1) pathway, cell growth, chemotaxis, angiogenesis, and vascular development. Suppressed genes were associated with neural growth factor (NGF) stimulated transcription, memory, nuclear events kinase and transcription factor activation, short-term memory, response to corticosterone, cell surface receptor signaling pathway involved in cell–cell signaling, ligand-activated transcription factor activity, and response to hypoxia. Network plots for the top differentially induced gene pathways in WT brains were axonogenesis, neuron projection guidance, developmental growth involved in morphogenesis, axon guidance, and developmental cell growth (Fig. 6C). The top differentially suppressed gene pathways included cognition, learning or memory, and muscle dell development (Fig. 6D).

We then performed a direct comparison of KO-O_2_ with WT-O_2_ animals. In this comparison, we found 1291 genes were differentially regulated in the dotplots, as illustrated in Fig. 7A. Genes induced by hyperoxia in GSDMD-KO brains relative to WT brains were associated with negative regulation of nervous system development, regulation of neuronal synaptic plasticity, hippocampus development, negative regulation of neurogenesis, endothelial cell chemotaxis, postsynapse organization, extracellular structure organization, and VEGFA/VEGFR2 signaling (Fig. 7B). Supressed genes included neurotransmitter receptors and postsynaptic signal transmission, neuroactive ligand receptor interaction, synaptic transmission, synapse asseembly, blood vessel diameter maintenance, vascular process in circulatiory system, reponse to BMP, and oligodendrocyte differentiation (Fig. 7B). Network plots for the top differentially induced gene pathways in the KO brains were regulation of neurogenesis, axonogenesis, synapse organization, dentate gyrus development, and limbi system development (Fig. 7C). Top suppressed gene pathways included regulation of blood circulation, glial cell differention, axon guidance, and myelination (Fig. 7D). These findings suggest that in the setting of hyperoxia, GSDMD-KO modulated important developmental pathways in the hippocampus.​

**Fig. 8:**
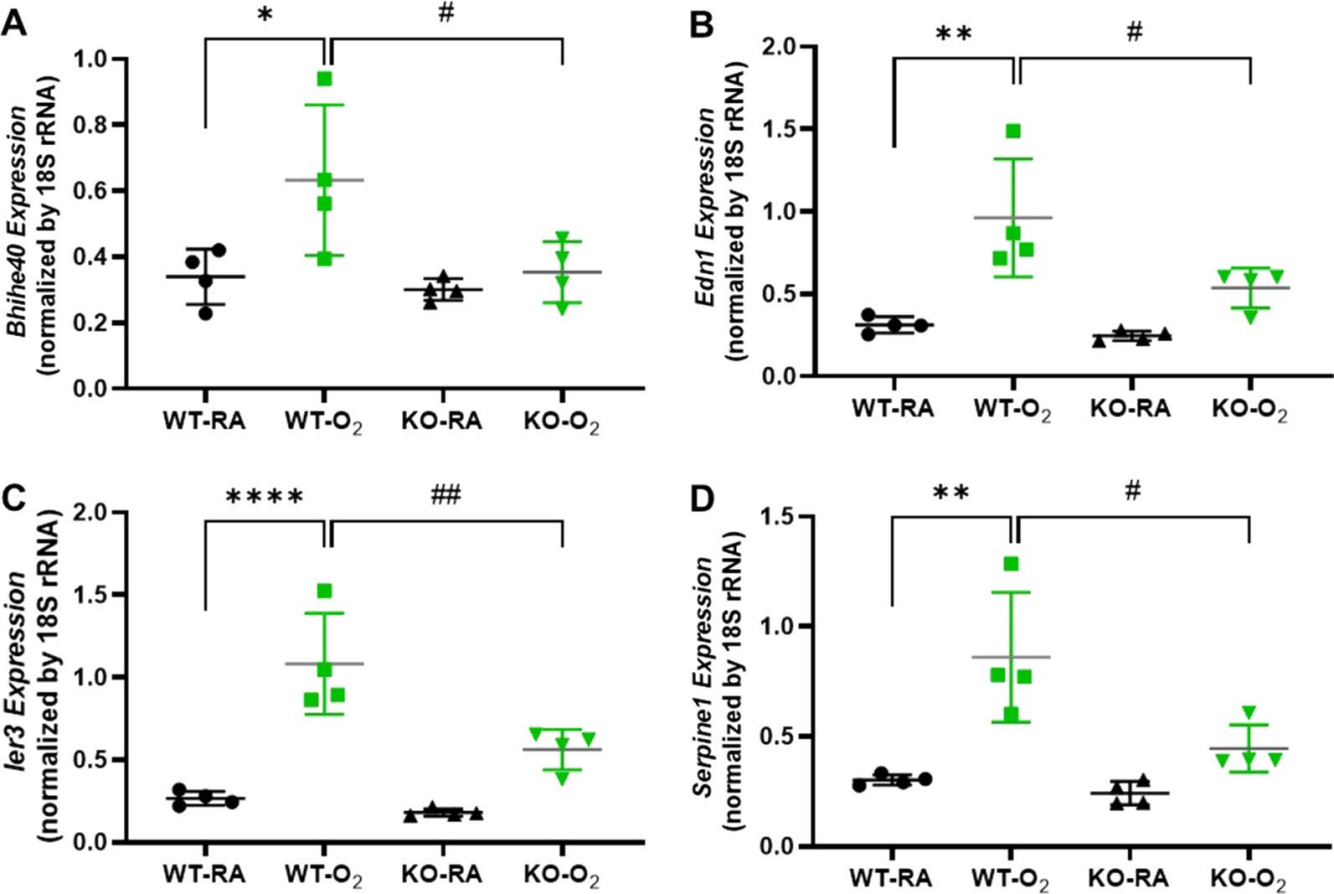
qRT-PCR validation of differentially regulated genes between WT-O_2_ and KO-O_2_ hippocampus. **A** Hyperoxia upregulated gene expressions of *Bhihe40*, *Edn1*, *ler3*, and *Serpine1* in the WT brains, but GSDMD-KO prevented hyperoxia upregulation of these genes. n = 4/goups. *P < 0.05, **P < 0.01, and ****P < 0.0001, WT-O_2_ compared to WT-RA. ^#^*P* < 0.05 and ^##^*P* < 0.01, KO-O_2_ vs. WT-O_2_

We performed qRT-PCR to verify select genes differentially regulated by hyperoxia and GSDMD-KO. Representative genes whose expression was increased by hyperoxia in the WT brains but reduced by GSDMD-KO included basic helix–loop–helix family member e40 (*Bhlhe40*), endothelin 1 (*Edn1*), immediate early response 3 (*Ier3*), and *Serpine1* which are involved in the neurovascular injury, synaptic plasticity, apoptosis, and cellular senescence (Fig. 8).

## Discussion

BPD, characterized by inflammatory lung injury, continues to be a major contributor to morbidity and mortality in extremely premature infants and is also a predictor of NDI [4, 5, 32]. Currently, no therapies are effective and safe for either condition. Previous studies from our lab have demonstrated a critical role for GSDMD activation in hyperoxia-induced mouse models of BPD and brain injury [15]. Furthermore, our previous studies also have shown that adoptive transfer of GSDMD-laden extracellular vesicle (EV) derived from hyperoxia-exposed rat models into healthy neonatal rats induced pathological hallmarks of BPD, and these GSDMD-laden EVs can cross the blood–brain barrier causing inflammatory brain injury [16]. In this study, we focused our investigations on the effects of global GSDMD-KO in a neonatal mouse model of hyperoxia-induced brain injury. We provided evidence, for the first time to the best of our knowledge, that GSDMD deficiency ameliorates hyperoxia-induced inflammation and cell death in the hippocampus as well as alters hyperoxia-modulated transcriptomes and distinctive enriched biological pathways in the hippocampus. However, since we used a global GSDMD KO model, it is likely that both KO of brain endogenous GSDMD expression as well as circulating EV GSDMD expression contribute to our observations. Both mechanisms can reduce GSDMD effects on brain inflammatory response and cell pyroptosis. Future studies are needed to differentiate these two mechanisms by generating lung cell-specific or brain cell-specific GSDMD gene deletion.

It is well-known that the etiology of lung injury and BPD in preterm infants is multifactorial. However, hyperoxia is thought to be a significant contributor to the inflammatory response mediated by macrophages and neutrophils, which invade the endothelium and alveolar spaces of premature lungs, causing lung injury and subsequent development of BPD [3]. A previous study from our laboratory demonstrated that hyperoxia-exposed GSDMD-KO animals had significantly less alveolar macrophage and neutrophil infiltration, an improvement in alveolarization and gas exchange surface area, improved vascularization and less vascular remodeling/muscularization compared to the WT mice indicating improvements in the hyperoxia-induced lung injury, and deranged alveolar and vascular development that are seen in BPD [21].

In addition, mounting evidence suggests hyperoxia is an important trigger of brain injury. Studies have shown that the developmental stages of the lung and brain in rodent models are comparable to preterm humans [33–35]. In most rodent models, lung injury has been noticed after exposure to hyperoxia for 7–14 days, whereas brain injury has been detected after exposure to hyperoxia for just 6 to 48 h. In our study, rodent models were exposed to hyperoxia for 14 days, aiming to investigate the chronic effects of hyperoxia on brain injury, and laying the foundation for further potential investigations of the complex lung–brain axis interactions which result in multiple comorbidities in preterm infants.

Our current study demonstrated a marked increase of GSDMD expression in the hippocampus of hyperoxia-exposed WT mice. We further showed that GSDMD was strongly expressed in microglial cells and astrocytes and weakly expressed in neural stem cells and progenitor cells. The strong expression of GSDMD in microglial cells was correlated with increased microglial cell activation and increased gene expression of inflammatory mediators, *Il6*, *Il1r1*, *Il18*, and *Il33*. These cytokines are known to be predominately produced by microglial cells, with basal amounts produced under steady-state conditions and greatly elevated amounts produced in response to pathogens, tissue damage, and other danger-associated molecular triggers, such as hyperoxic tissue damage [36]. They play pivotal roles in CNS infection, neurodegeneration, and injury. Our results support the crucial role of GSDMD in mediating hyperoxia-induced brain inflammation.

Based on the evidence of GSDMD involvement in the inflammasome pathway, we investigated the effects of GSDMD-KO on cell proliferation and death. Although cell proliferation was generally low in all study groups, the GSDMD-KO brain had a higher proliferating index than WT brain under hyperoxic conditions. Based on the location of these proliferating cells in the subgranular zone (SGZ) of the dentate gyrus as well as the cornu ammonis and surrounding hippocampus, these cells are likely neural stem cells and progenitor cells that are known to be present and proliferating in the neonatal hippocampus. Our results demonstrated that hyperoxia-induced hippocampal cell death was significantly lower in the GSDMD-KO mice compared to their WT littermates. Furthermore, the TUNEL-positive cells were mainly co-localized with neural stem cells, progenitor cells, and astrocytes in hyperoxia-exposed WT brains. These results highlight a detrimental effect of hyperoxia on neural stem cell physiology and function, which can lead to short-term and long-term neurodevelopmental impairment, as observed in preterm infants with a history of chronic oxygen therapy [4].

Our RNA-seq findings reveal that hyperoxia-induced structural damage is associated with the altered expression of many gene pathways that can impact brain development in WT mice but not in GSDMD-KO mice. There was a clear separation of WT and KO mice by PC1, and WT-RA vs. WT-O_2_ on PC3. However, there was no clear separation of KO-RA vs. KO-O_2_ in PC3, indicating that GSDMD-KO prevents hyperoxia-induced transcriptome changes in the mouse brain. These observations were further supported by the distinct heatmap of differentially expressed genes and hierarchical clustering of genes by genetic backgrounds and conditions in WT-O_2_ vs. WT-RA and KO-O_2_ vs. KO-RA animals.

On differential expression analysis, hyperoxia differentially regulated 258 genes, with 146 genes upregulated and 112 genes downregulated in the WT animals. However, hyperoxia only differentially regulated 16 genes in the GSDMD-KO animals. On overrepresentation analysis of the WT brains, hyperoxia-induced genes are associated with neuroprotection morphogenesis, neuron differentiation and development, axonogenesis, blood circulation, HIF-1 pathway, cell growth, chemotaxis, angiogenesis, and vascular development. Suppressed genes were associated with NGF-stimulated transcription, memory, transcription factor activation, short-term memory, response to corticosterone, cell surface receptor signaling pathway involved in cell–cell signaling, ligand-activated transcription factor activity, and response to hypoxia. However, these changes were prevented by GSDMD-KO, which led to the regulation of fewer genes that were not associated with these processes or pathways, suggesting they are GSDMD-dependent. HIF-1 signaling pathways are critically involved in the embryonic and postnatal stages of brain development by targeting the highly active maturational and angiogenic processes [37]. VEGF is known to be a major target gene for HIF-1. Hyperoxia-induced destabilization of HIF-1 downregulates the expression of proangiogenic factors, such as VEGF and its receptors VEGFR1 and VEGFR2, leading to derangement of angiogenesis and vascular development [38, 39]. NGF is a neurotrophic factor that plays a central role in the growth, development, and protection of the central and peripheral nervous systems [40, 41] and is, therefore, critical for prenatal and postnatal brain development. Preventing its downregulation by hyperoxia by GSDMD-KO highlights a crucial function of GSDMD in neonatal brain development.

When directly comparing KO-O_2_ with WT-O_2_, we found 1291 genes were differentially regulated, with genes associated with negative regulation of nervous system development, regulation of neuronal synaptic plasticity, hippocampus development, negative regulation of neurogenesis, endothelial cell chemotaxis, postsynapse organization, extracellular structure organization, and VEGFA/VEGFR2 signaling being upregulated. Upregulation of these important neurodevelopmental pathways by GSDMD-KO indicates they are GSDMD responsive under hyperoxia exposure, which may lead to better brain development in neonatal mice. Further upregulation of VEGFA/VEGFR2 signaling by GSDMD-KO may facilitate neurovascular development, which is important for brain function. We discovered some down-regulated gene pathways by GSDMD-KO that were associated with neurotransmitter receptors and postsynaptic signal transmission, neuroactive ligand receptor interaction, synaptic transmission, synapse assembly, blood vessel diameter maintenance, vascular process in circulatory system, reponse to BMP, and oligodendrocyte differentiation. We speculate changing in these pathways may provide balance for normal brain development under hyperoxia. Our network plotting data further support the critical role of GSDMD in modulating important neurodevelopmental pathways in the hippocampus.

We reported four representative genes whose expression was increased by hyperoxia in WT brains but reduced by GSDMD-KO, which included *Bhlhe40*, *Edn1*, *Ier3*, and *Serpine1*. *Bhlhe40* is a transcription factor that: (1) directly represses gene expression via binding to class B E-Box sequences (CACGTG) [42]; (2) directly activates gene expression by binding to Sp1 sites [43, 44]; and (3) indirectly regulates gene expression by interacting with basal transcription machinery, other transcription factors, or histone modifiers [45–47]. It is highly expressed in the hippocampus and involved in a number of essential functions, such as hypoxia, DNA damage responses, and metabolism [45, 48–50]. It plays a role in regulating neuronal excitability and synaptic plasticity in the hippocampus [51]. *Edn1* is involved in regulating neurotransmission, microglial proliferation, and maintenance, and EDN1–endothelin receptor B complex contributes to oligodendrocyte differentiation and myelin deficits during preterm white matter injury [52–54]. *Ier3* gene is involved in regulating apoptosis in various organs [55, 56], and its role in preterm brain injury is unknown. *Serpine1*, also known as plasminogen activator inhibitor-1 (PAI1) is involved in regulating cellular senescence and neuroinflammation [57, 58]. *Serpine1* acts as a regulator of peripheral neutrophil migration, independent of its role as a protease inhibitor, contributing to ischemic stroke [59]. These findings further demonstrate that the genes and the corresponding pathways regulated by hyperoxia could be prevented by GSDMD-KO, thereby alleviating inflammatory responses, cell death, and vascular and tissue remodeling in premature brains.

We conclude that deficiency of GSDMD largely attenuates the damaging effects of hyperoxia on the premature brain at structural and cellular levels, which are linked to transcriptome modifications. GSDMD-KO resulted in the upregulation of gene pathways related to neuronal synaptic plasticity, hippocampus development, neurogenesis, endothelial cell chemotaxis, postsynapse organization, extracellular structure organization, and VEGFA/VEGFR2 signaling. GSDMD-KO resulted in downregulating gene pathways associated with neurotransmitter receptors and postsynaptic signal transmission, neuroactive ligand receptor interaction, synaptic transmission and synapse assembly, blood vessel diameter maintenance, and oligodendrocyte differentiation. Although the cellular sources for these transcriptome modifications are unknown due to the limitations of whole hippocampal RNA-seq, which lacks the ability to detect gene expression in specific brain cells compared to single-cell RNA-seq, which can detect hyperoxia-induced gene changes in specific brain cells. We plan to perform single-cell RNA-seq in future studies to expend and confirm our whole hippocampal RNA-seq data.

The results from this study, combined with our recently published data on the effects of GSDMD deficiency in ameliorating hyperoxia-induced lung and retinal injury in neonatal mice [21], highlight that the inflammasome–GSDMD cascade is central to hyperoxia-induced premature multi-organ injury. Thus, targeting GSDMD may be beneficial in preventing and treating neonatal brain, lung, and retinal damage in premature infants.

### Supplementary Information

**Additional file 1:** List of materials.

## Data Availability

The data sets generated during and/or analyzed during the current study are available from the corresponding author upon reasonable request. RNA-seg data could be assessed by GEO series number GSE241549.
