# Supplementary material for: GSDMD gene knockout alleviates hyperoxia-induced hippocampal brain injury in neonatal mice
Source: J Neuroinflammation. 2023 Sep 7;20:205. doi: 10.1186/s12974-023-02878-8 (PMC10486051; doi:10.1186/s12974-023-02878-8)
Supplement: Supplementary file 1 — Additional file 1: List of materials. [file 12974_2023_2878_MOESM1_ESM.docx]

**Online Supplement**

| **Antibody list** | **Company** | **Item number** | **Usage** |
| --- | --- | --- | --- |
| Goat anti-AIF-1 | NovusBio | NB100-0128 | Immunostaining (2.5 μg/ml)  Immunofluorescence (5.0 μg/ml) |
| Rabbit anti-CD68 | Cell Signaling | E307V | Immunostaining (0.25 μg/ml) |
| Mouse anti-GFAP | BioLegend | 801103 | Immunofluorescence (2 μg/ml) |
| Rabbit anti-GSDMD | Abcam | ab219800 | Immunostaining (10 μg/ml)  Immunofluorescence (10 μg/ml) |
| Rabbit anti-Ki67 | Abcam | ab15580 | Immunofluorescence (10 μg/ml) |
| Mouse anti-NeuN | Abcam | ab104224 | Immunofluorescence (1 μg/ml) |
| Goat anti-SOX2 | R&D Biosystems | 967242 | Immunofluorescence (4 μg/ml) |
|  |  |  |  |
| **PCR primer list** | **Company** | **Item number** | **Usage** |
| Il11β | ThermoFisher | Mm00434228_m1 | qRT-PCR |
| Il1r1 | ThermoFisher | Mm00434237_m1 | qRT-PCR |
| Il18 | ThermoFisher | Mm00434226_m1 | qRT-PCR |
| Il33 | ThermoFisher | Mm00505403-m1 | qRT-PCR |
| Il6 | ThermoFisher | Mm00446190_m1 | qRT-PCR |
| Edn1 | ThermoFisher | Mm00438659_m1 | qRT-PCR |
| Ier3 | ThermoFisher | Mm00519290_g1 | qRT-PCR |
| Bhlhe40 | ThermoFisher | Mm00478593_m1 | qRT-PCR |
| Serpine1 | ThermoFisher | Mm00435858_m1 | qRT-PCR |
|  |  |  |  |
| **Other reagents** | **Company** | **Item number** | **Usage** |
| miRNeasy Mini Kit | Qiagen | 217004 | RNA isolation |
| TUNEL assay | ThermoFisher | C10245 | Detection of apoptotic cells |
